# Airway Transepithelial Transport of IgG Formulated with Excipients: A Case Study on What to Consider Regarding Impact on Epithelial Barrier Integrity, IgG Quality and Matrix Effects During the Analytics

**DOI:** 10.3390/pharmaceutics18101262

**Published:** 2026-10-06

**Authors:** Rebecca Rittersberger, Gabriel Koslowski, Iman Shrimo, Katharina Schindowski

**Affiliations:** 1 Institute of Applied Biotechnology, University of Applied Science Biberach, Hubertus-Liebrecht Strasse 35, 88400 Biberach, Germany; 2 Faculty of Natural Science, University of Ulm, Albert-Einstein-Allee 11, 89081 Ulm, Germany

**Keywords:** biopharmaceutical formulation, excipients, polysorbate, cyclodextrin, poloxamer, biopharmaceutical stability, primary cell model, toxicity, transcytosis, mucosal drug delivery, inhalation

## Abstract

**Background**: Surface-active excipients such as polysorbate 20 (PS20), polysorbate 80 (PS80), poloxamer 188 (Px188), and cyclodextrin (CD) are widely used to stabilize IgG formulations, including aerosols. However, their effects on transepithelial IgG transport and the reliability of associated analytical readouts remain poorly understood. **Methods**: Transepithelial transport of Bevacizumab was quantified across a primary airway epithelial model in the presence of clinically relevant concentrations of PS20, PS80, Px188, or CD using ELISA. Potential methodological bias was assessed by cytotoxicity and barrier integrity (Alamar Blue, LDH, TEER), IgG monomer content (SE-HPLC), micelle formation and excipient passage, and ELISA matrix effects using excipient-containing calibration curves. **Results**: Only CD at 10 mg/mL significantly increased IgG transport, whereas PS20, PS80, and Px188 had no significant effect. All excipients were non-toxic, with TEER being the most sensitive indicator. IgG monomer content remained unchanged under all conditions. Micelle-forming activity of PS20, PS80, and CD was retained, but only CD (10 mg/mL) crossed the epithelial barrier (11% of the initial concentration). Excipients caused concentration-dependent ELISA matrix effects; however, matrix-adjusted quantification showed only a modest, non-significant change in calculated IgG transport for CD. **Conclusions**: Excipients can influence in vitro IgG transport studies through biological and analytical mechanisms. Under the tested conditions, the increased IgG transport observed with CD appears to be genuine rather than assay-related, highlighting the importance of controlling excipient-related effects in mucosal IgG transport studies.

## 1. Introduction

The delivery of therapeutic immunoglobulin G (IgG) as an aerosol enables the targeting of the airways from intranasal nose-to-brain up to inhalative delivery [1,2,3,4]. Targeted mucosal administration allows for both local and systemic therapeutic effects and is suitable for outpatients’ therapy, improves patient adherence and is cost-effective for healthcare services [5,6]. However, several studies have shown that IgG is prone to aggregate during aerosolization [7,8,9,10,11]. Therefore, the complex structure of IgG-based therapeutics requires an individual formulation strategy with excipients to sustain the IgG’s stability during aerosolization [7].

Excipients such as surfactants (e.g., polysorbates and poloxamers) and solubilizing agents (e.g., cyclodextrins) are routinely used to stabilize proteins during production, storage, and administration [12,13,14]. The surface-active excipients Polysorbate 20 (PS20), Polysorbate 80 (PS80), Poloxamer 188 (Px188), and 2-Hydroxypropyl-β-cyclodextrin (CD) are commonly used in stabilizing biopharmaceutical formulations [12,13,14] and their mechanisms are well described [13]. In aerosol-based drug delivery, these excipients reduce protein aggregation or denaturation at the air–liquid interface but can also have the opposite effect if the formulation is not optimized [7,9,15,16,17,18,19]. However, hydrophilic macromolecules like many biopharmaceuticals have limited uptake across (mucosal) barriers due to their molecular size and physicochemical properties [20]. For both systemic and local therapies (e.g., anti-infective antibody treatments), API uptake is crucial for efficacy. Prior studies on the excipients’ effect on drug uptake mostly focused on small molecules or peptides [21], used different excipients [22], cell types [23,24], or formulations [24].

As the vast majority of previous studies investigated the uptake and permeability of IgG at airway mucosa models mostly in phosphate-buffered saline (PBS), some of them added amino acids for stabilization but without any of the above-mentioned excipients; therefore, the effects of these excipients on drug uptake and quantification are not yet sufficiently described [2,25,26,27,28,29]. While in powder-dried or powder-sprayed protein aerosols, the use of excipients is well detailed [30,31], only a few studies used excipients to stabilize liquid protein aerosols [7,32]. While the stabilizing properties of excipients are well documented, published data suggest that they may influence analytical quantification, and even drug uptake [7,33,34,35,36,37,38,39,40,41].

Therefore, we investigated in the present study the impact of the excipients PS20, PS80, Px188, and CD on transepithelial IgG transport in an air–liquid interface (ALI) primary airway model and on ELISA-based IgG quantification, their toxicity, impact on IgG quality and their potential to cross the epithelial barrier. Our findings highlight the need to validate analytical methods in the presence of excipients and underscore the multifaceted role of formulation in the development of inhalable biopharmaceuticals.

## 2. Materials and Methods

### 2.1. Materials

Polysorbate 20 (PS20; Carl Roth, Karlsruhe, Germany), polysorbate 80 (PS80; Carl Roth, Karlsruhe, Germany), 2-Hydroxypropyl-β-cyclodextrin (CD; AppliChem, Darmstadt, Germany) and poloxamer 188 (Sigma-Aldrich, Darmstadt, Germany) were used as investigated excipients. For immunoglobulin G (IgG), an in-house-produced biosimilar of Bevacizumab was used. All excipients were diluted in phosphate-buffered saline (PBS) pH 7.4 or MEM medium. The osmolality of excipients used in this study is displayed in Supplementary Table S1.

### 2.2. Cell Culture

#### 2.2.1. Tracheal Mucosal Primary Cells (TMPCs)

The primary cells were isolated from tracheal tissue collected post mortem from pigs slaughtered for food production and approved for human consumption after official veterinary inspection. No animals were used, treated, or killed for scientific purposes. Ethical approval was not required for the use of these materials under German national regulations. TMPCs were obtained from 4- to 6-month-old pigs from a local slaughterhouse. The mucosa from the trachea was excised and subjected to further steps within 1–2 h post mortem. In the first step, the lamina propria and the respiratory epithelium were removed from the hyaline cartilage. Subsequently, the mucosal samples were disinfected using Octenisept^®^ (Schülke & Mayr GmbH, Norderstedt, Germany) and were washed twice with prewarmed PBS (Carl Roth, Karlsruhe, Germany). After that, the samples were transferred into T25 flasks, 20 mL pronase medium (1.4 mg/mL Pronase (Sigma-Aldrich, Taufkirchen, Germany) in EBSS (Thermofisher Scientific, Darmstadt, Germany) + 20 U/mL penicillin, 20 µg/mL streptomycin (both Thermofisher Scientific, Darmstadt, Germany) + 200 µg/mL Gentamycinsulfate + 200 µg/mL Kanamycinsulfate + 5 µg/mL Amphotericin B (all from Carl Roth, Karlsruhe, Germany)) was added, and the flask was incubated at 37 °C, 5% CO_2_, and 85% relative humidity (rH) for 1 h. Afterwards, the supernatant containing TMPCs was removed and processed with debris removal solution (Miltenyi Biotec, Bergisch Gladbach, Germany) according to the manufacturer`s instructions. The cells were directly seeded into membrane inserts (1 µm pore size, Greiner BioOne, Kremsmünster, Austria) with a cell density of 10^5^ cells/insert. Coating of all surfaces was performed with 0.05 mg/mL rat tail collagen type I (Primacyt, Schwerin, Germany) solution at least 2 days before cultivation. After 24 h, the cells were lifted to air–liquid interface (ALI) conditions by removing the apical medium and exchanging the basolateral medium (DMEM:F12 (1:1) (Th. Geyer, Renningen, Germany) + 20% FBS (Capricorn, Ebsdorfergrund, Germany) + 2 mM Glutamine (Biowest, Nuaillé, France) + 4.5 g/L Glucose (Carl Roth, Karlsruhe, Germany) + 1% NEAA + 20 U/mL penicillin, 20 µg/mL streptomycin (all from Thermofisher Scientific, Darmstadt, Germany) + 200 µg/mL Gentamycinsulfate + 200 µg/mL Kanamycinsulfate + 5 µg/mL Amphotericin B (all from Carl Roth, Karlsruhe, Germany). The cells were cultivated for 21 days with a washing step with sterile PBS pH 7.4 and a medium exchange every 2 days. A detailed workflow has been published elsewhere [1].

#### 2.2.2. RPMI 2650 Nasal Airway Cells

RPMI 2650 is a cell line exhibiting epithelial morphology from the nasal septum of a 52-year-old male patient with squamous cell carcinoma (ATCC, American Tissue and Culture Collection). RPMI 2650 cells were cultivated in T-flasks at 37 °C, 5% CO_2_, and 85% rH using MEM (Th. Geyer, Renningen, Germany) supplemented with 10% FBS (Capricorn, Ebsdorfergrund, Germany), 2 mM Glutamine (Biowest, Nuaillé, France), 10 U/mL Penicillin and 10 μg/mL Streptomycin (Thermofisher Scientific, Darmstadt, Germany) until they reached 80–90% confluency. Antibiotics were only used when cells were cultivated in membrane inserts. Cells were washed twice with prewarmed PBS and detached by trypsinization using trypsin/EDTA solution (Capricorn, Ebsdorfergrund, Germany) from the T-flasks. RPMI 2650 cells were used up to passage 25 for seeding into membrane inserts with a cell density of 10^5^ cells/insert. For RPMI 2650, the same membrane inserts and differentiation conditions were used as described for TMPCs in Section 2.2.1.

### 2.3. Transepithelial Electrical Resistance (TEER) Measurement

We utilized TEER measurement to test for an adequate barrier function of the TMPCs before starting the transport experiments with IgG, but also as an additional cytotoxicity readout. The cultivation medium was removed from the membrane inserts and 350 μL MEM w/o phenol red (Thermofisher Scientific, Darmstadt, Germany) was added apically, and 500 μL MEM w/o phenol red was added to the basolateral compartment. Inserts were placed in the incubator for 10 min at 37 °C, 5% CO_2_, and 85% rH, followed by another 10 min incubation at room temperature to let cells equilibrate. The resistance was measured using the EVOM3 device and chopstick electrodes (World Precision Instruments, Sarasota, FL, USA). The blank membrane insert without cells was subtracted from the raw data and multiplied by the growth area of the membrane inserts (0.336 cm^2^).

### 2.4. Transport Experiments of IgG with Different Surface-Active Excipients

Primary cells were only used for transport experiments if the TEER value was above 500 Ω × cm^2^ as described previously [1,42]. MEM w/o phenol red (from TEER measurement) was removed from the apical and basolateral compartments, and 260 μL of medium (MEM w/o phenol red) + 20 U/mL penicillin, 20 µg/mL streptomycin (all from Thermofisher Scientific, Darmstadt, Germany) + 200 µg/mL Gentamycinsulfate + 200 µg/mL Kanamycinsulfate + 5 µg/mL Amphotericin B (all from Carl Roth, Karlsruhe, Germany) was added to the basolateral compartment. Adapted from previous studies [26], 100 μL of 500 µg/mL Bevacizumab, either in PBS only as a non-formulated control or in PBS containing one of the four different surface-active excipients in a concentration as indicated, was added to the apical compartment of the TMPC membrane inserts. The total volume of the basolateral compartment was collected after 24 h for IgG quantification by ELISA.

### 2.5. Enzyme-Linked Immunosorbent Assay (ELISA)

An Fc-Fc ELISA was performed to quantify the transepithelial transport of IgG. This type of ELISA is based on immunodetection of human IgG by using two human Fc region-specific antibodies. A high-binding 96-well plate (Thermofisher Scientific, Darmstadt, Germany) was coated with 100 μL of a 1:7500 dilution of the capture antibody in a 100 mM bicarbonate buffer pH 9.6 (coating buffer; Carl Roth, Karlsruhe, Germany) of the capture antibody (#I2136; Sigma-Aldrich, Darmstadt, Germany). The plate was then incubated overnight at 4 °C. After coating, the plate was washed three times for 3 min with 200 μL PBS supplemented with 0.05% Tween 20 (PBST; Tween 20 is a commercial brand name for polysorbate 20; Carl Roth, Karlsruhe, Germany). For blocking, 100 μL blocking solution (PBS with 1% BSA; Carl Roth, Karlsruhe, Germany) was added to each well and incubated for 2 h at 37 °C or overnight at 4 °C. After blocking, the plate was washed four times for 3 min with 200 μL PBST before 100 μL of each sample diluted (1:200 or 1:1000) in MEM was added. The plate was then incubated overnight at 4 °C and washed the next day four times again for 3 min with 200 μL PBST. The detection antibody (#A0170; Sigma-Aldrich, Darmstadt, Germany) was diluted 1:50,000 in PBS; 100 μL per well was added and incubated for at least 2 h at 37 °C. The final washing step was performed as described above. For colorimetric evaluation, 100 μL TMB (3,3′,5,5′-Tetramethylbenzidin; Thermofisher Scientific, Darmstadt, Germany) was added to each well. Next, the plate was placed in the incubator for 10–30 min at 37 °C. Finally, 50 μL of 1 M HCl (Bernd Kraft GmbH, Oberhausen, Germany) was added to each well to stop the reaction. Absorbance was measured at 450 nm and concentrations in the samples were interpolated with GraphPad Prism 10.6.1 software (GraphPad Software, Boston, MA, USA).

As standards for the initial experiment, Bevacizumab was titrated in PBS. To analyze how excipients influence the ELISA standard curves, Bevacizumab was titrated in PBS containing the indicated excipients.

### 2.6. Cytotoxicity Assays

#### 2.6.1. Alamar Blue

This assay determines the oxidizing activity of mitochondria of viable, healthy cells by reducing the non-fluorescent resazurin to fluorescent resorufin [43,44]. The Alamar Blue assay (Thermofisher Scientific, Darmstadt, Germany) was performed according to the manufacturer’s instructions. In brief, 100 µL of the different excipient samples was added apically to the TMPCs or RPMI 2650 cells grown on membrane inserts for 21 days under ALI conditions. As a non-toxic negative control, MEM w/o phenol red (Thermofisher Scientific, Darmstadt, Germany) was used and in the later evaluation set to 100% viability. Furthermore, 50% DMSO (Carl Roth, Karlsruhe, Germany) was applied as a positive control for cytotoxicity. After an incubation for 24 h at 37 °C, 5% CO_2_ and 85% rH, the basolateral medium was exchanged, and the samples were removed and replaced by 100 µL of a 1:10 dilution of the Alamar Blue reagent in MEM w/o phenol red for 5 h. Afterwards, the apical volume was removed, and the fluorescence was measured with 530 nm for excitation and 590 nm for emission. A solution was declared cytotoxic if the viability after treatment was below 70% compared to the MEM control. This assumption derives from an adapted version of the “Test for in vitro cytotoxicity” (ISO 10993-5), which defines a substance as “non-cytotoxic” if the viability after treatment is above 70% [45].

#### 2.6.2. Lactate Dehydrogenase (LDH) Release Assay

In this assay, LDH that is released by damaged cells catalyzes the conversion of lactate to pyruvate via NAD+ reduction to NADH. Oxidation of NADH by diaphorase reduces the tetrazolium salt iodonitrotetrazolium (INT) to a red formazan product [46]. The LDH assay (Thermofisher Scientific, Darmstadt, Germany) was performed according to the manufacturer’s instructions. Cell exposure with the excipients containing samples was performed as in 2.4. After 24 h, the basolateral medium was used for the determination of released LDH. Furthermore, 10 mg/mL PS20, which lyses cell membranes at these high concentrations, served as a positive control and was set to 0% viability, and MEM medium served as a negative control. A total of 50 µL of each sample was mixed with 50 µL of reaction buffer and incubated for 30 min. Absorbance was measured at 490 and 680 nm. Again, a solution was declared cytotoxic if the viability after treatment was below 70% compared to the MEM control [45].

### 2.7. IgG Stability Analysis via Size-Exclusion Chromatography

The different formulations of IgG were analyzed via size-exclusion–high-performance liquid chromatography (SE-HPLC) directly after preparation and 24 h after incubation at 37 °C, 5% CO_2_ and 85% relative humidity. The analysis was performed at an Agilent 1100 HPLC (Agilent, Santa Clara, CA, USA) with an AdvanceBio SEC column (300 Å, 7.8 × 300 mm, 2.7 µm; Agilent, Santa Clara, CA, USA). Detection wavelength was set to 220 nm. PBS pH 7.4 (Carl Roth, Karlsruhe, Germany) was used as elution buffer at a flow rate of 0.5 mL/min at 20 °C. Samples were filtered with a 0.45 µm filter (Pall GmbH, Dreieich, Germany before analysis and were compared for the monomer peak area under the curve (AUC).

### 2.8. Fluorescence Micelle Assay (FMA)

FMA was utilized to determine the stability of the micelles formed by the excipients (PS20, PS80 and CD) under the following conditions: (i) freshly prepared dilutions of excipients containing 0.5 mg/mL IgG in PBS as control (set as 100% micelle formation for each excipient), (ii) samples from the above-mentioned control stored for 24 h at 37 °C in the cell culture incubator, (iii) samples from diluted excipients containing 0.5 mg/mL IgG in PBS that had been applied to the apical compartment of the transwell membrane inserts grown with TMPC and incubated at 37 °C, 5% CO_2_ and 85% rH for 24 h and (iv) samples from the basolateral compartment after 24 h exposure of the indicated excipients at the apical side indicating the content of micelle-forming levels that can be recovered from the basolateral side. As a negative control (set to 0%) 0.5 mg/mL IgG in PBS was used in the FMA. For the measurement, 10 μL of the above-mentioned samples was mixed with 240 μL FMA reagent (150 mM NaCl (Carl Roth, Karlsruhe, Germany), 50 mM Tris (Carl Roth, Karlsruhe, Germany), 0.2% acetonitrile (Carl Roth, Karlsruhe, Germany), 5 μM N-phenyl-1-naphtylamine (NPN, Thermofisher Scientific, Darmstadt, Germany), 0.0015% Brij-35 (Carl Roth, Karlsruhe, Germany), pH 8) and incubated for 1 min at 35 °C [47]. During incubation, the fluorescent dye NPN intercalates into excipient-formed micelles. Fluorescent signal, correlating with excipient concentration, was measured at 420 nm following excitation of the dye with a 350 nm laser using an Infinite^®^200 PRO microplate reader (Tecan, Männedorf, Switzerland). Samples from the basolateral compartment were corrected to a 260 µL volume.

### 2.9. Statistical Analysis, Illustrations and Language Correction

All results are presented as mean ± standard deviation (SD) unless otherwise stated. If not stated otherwise, n equals technical replicates, like individual membrane inserts, and N equals independent experiments. Graphs were created and statistical evaluation was performed using GraphPad Prism 10.6.1 software (Boston, MA, USA). Biostatistical tests are indicated. Illustrations were created under licence with https://www.biorender.com. For English language correction and surface-level text editing, the language models Ask Alma (based on OpenAI, version 5.1; KerstaGmbH, Welzheim, Germany) and ChatGPT (OpenAI, CA, USA; version 5) were used, and all authors verified the intended meaning was retained. Generative artificial intelligence was not used for any other purposes than text correction and editing.

## 3. Results

**Figure 1:**
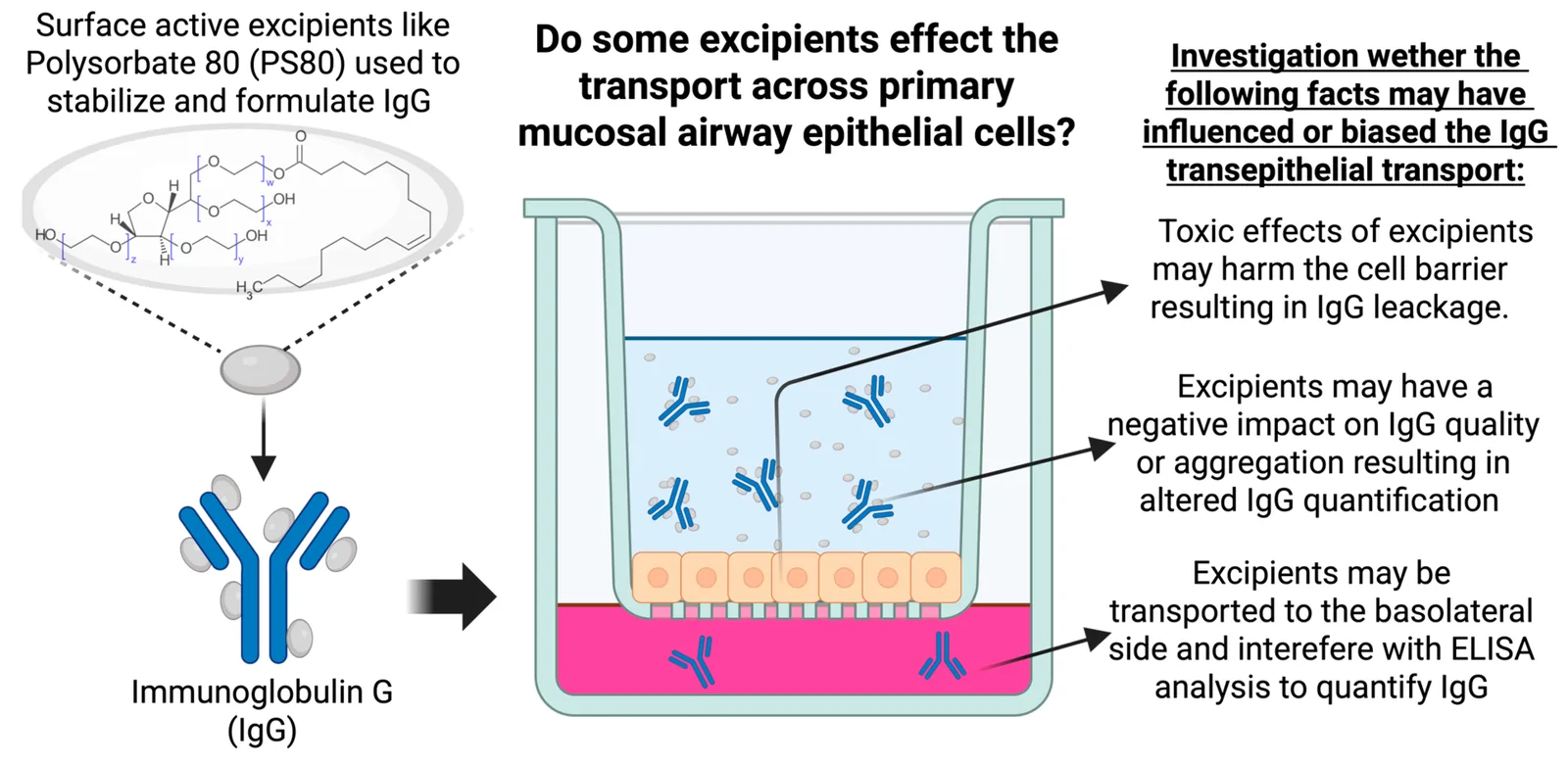
Methodological investigation to identify factors for potential false-positive or false-negative results when analyzing transepithelial IgG (Bevacizumab) transport in the presence of the excipients PS20, PS80, Px188, or CD used commonly in clinical IgG formulations. Since certain aspects may influence the transport in a primary epithelial model derived from porcine trachea, the following analyses were done: Any cytotoxic effects of the excipients were evaluated and the non-toxic range was determined to avoid any impact and false-positive results on IgG transport. The impact of the above-mentioned excipients on stabilizing the IgG monomer content as a representative of IgG quality under cell culture conditions was evaluated. The activity of excipients to form micelles under cell culture conditions and when exposed to the apical surface of airway epithelial cells was assessed. Also, it was investigated whether detectable concentrations of excipients could be recovered from the basolateral compartment as they may interfere with ELISA quantification of IgG by matrix effects. Finally, the impact of a (potential) presence of excipients during ELISA was assessed and how this impacts the outcome in the transport studies. Created in BioRender. Zimmermann, K. (2026) https://BioRender.com/2kydkxo.

The commonly used excipients PS20, PS80, Px188, and CD have demonstrated their potential in protein stabilization for IgG aerosols [7,9], but their influence on IgG uptake and transport is hardly reported. In addition, the presence of surface-active substances may interfere with the analytical quantification of IgG [48]. Hence, we investigated here potential methodological causes or bias for our unpublished observations that the transepithelial transport of a monoclonal IgG (Bevacizumab biosimilar) could be altered in the presence of excipients. As excipients we used the very well-established molecules used in IgG formulations: PS20, PS80, Px188, and CD. Transport studies were performed in a primary airway epithelial cell model [1]. Excipients can cause different false-positive or false-negative scenarios that have the potential to impact or interfere with transport or the associated analysis for IgG quantification (Figure 1):

- Like all molecules, excipients can exhibit toxic effects on (primary) epithelial cells when present in sufficient concentration, resulting in a loss of their barrier integrity and/or cell death. Such loss of the cell layer’s integrity results in leakage and, thus, apically applied IgG can more easily cross the leaky cell layer to the basolateral compartment than under intact conditions.
- Any impact on IgG’s quality and structure caused by excipients may interfere with IgG quantification by ELISA.
- Excipients may also cross the intact epithelial barrier to the basolateral compartment. Their presence may additionally interfere with IgG quantification by ELISA, known as a matrix effect.

Verification or exclusion of the above-mentioned causes is important to unravel the real impact of excipients on transepithelial IgG transport.

### 3.1. Transepithelial Transport of IgG with Different Surface-Active Excipients: Is It a True or Biassed Result?

**Figure 2:**
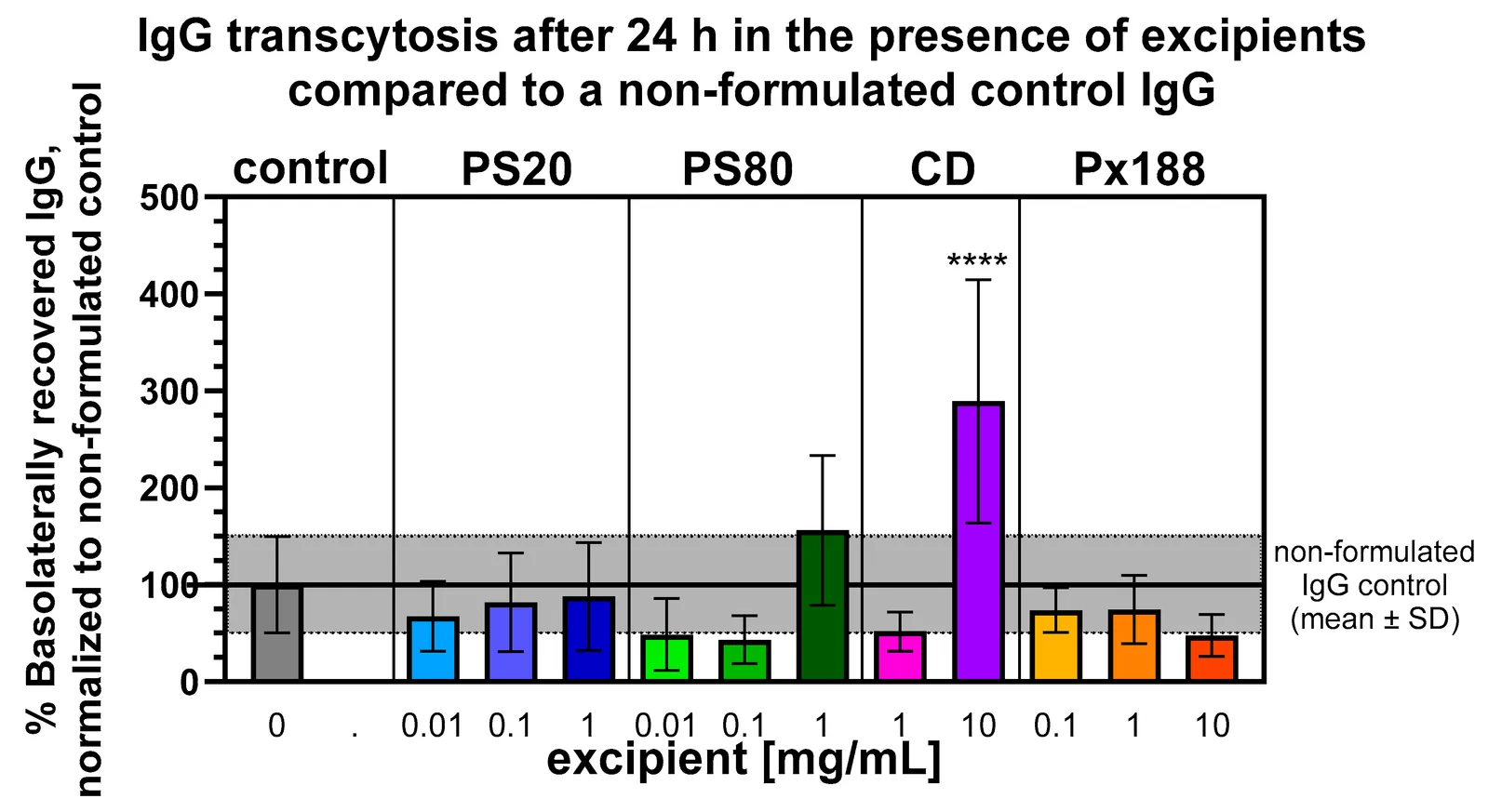
Effect of the excipients PS20, PS80, CD and Px188 on transepithelial transport of IgG. The non-formulated IgG control in PBS without any excipient was set to 100% and all other transport rates determined in the presence of the indicated excipients after 24 h through the TMPC model were calculated accordingly. The mean of the non-formulated control is displayed as black solid line and the ± SD is depicted as a grey background. Excipients were used in the indicated concentrations [mg/mL]. Only 10 mg/mL CD increased the IgG transport significantly (**** *p* ≤ 0.0001; one-way ANOVA with Dunnett’s multiple comparison); all other different concentrations of the excipients clearly failed to reach statistical significance. All data are displayed as mean ± SD.

We investigated transepithelial transport with Bevacizumab in a previously published primary airway epithelial (TMPC) model [1,49] with the excipients PS20 (used at 0.01, 0.1, and 1 mg/mL), PS80 (used at 0.01, 0.1, and 1 mg/mL), CD (used at 1 and 10 mg/mL), and Px188 (used at 0.1, 1, and 10 mg/mL) compared to an unformulated control in PBS without any additional excipients. IgG quantification from the basolateral compartment was performed with a standard ELISA calibration from Bevacizumab titrated in MEM medium as matrix of the basolateral compartment without any excipients. The outcome of this study is displayed in Figure 2.

The presence of 10 mg/mL CD in PBS significantly elevated basolateral IgG levels (**** *p* ≤ 0.0001; 3422.1 ± 1482.39 ng) compared to the non-formulated control in PBS (1182.35 ± 586.41 ng; one-way ANOVA with Dunnett’s multiple comparisons test; N = 3; n = 6). Although the addition of 1 mg/mL PS80 appeared to result in increased basolateral levels of 1847.7 ± 913.65 ng, this was statistically not significantly altered compared to the non-formulated control (*p* = 0.39). The original data for the total mass of IgG recovered from the basolateral compartment, as well as the data expressed as a percentage of the apically applied dose, are provided in Supplementary Figure S1.

In a previous study from our lab, a formulation containing both PS20 and CD enhanced transport of a Fab fragment—but not of a full IgG in a delivery model for inhalation [7]. That study used the RPMI2650 cell line, which is more permeable than the TMPCs used here [1,50] and applied lower CD concentrations (2–3.5 mg/mL). Proposed mechanisms for CD-enhanced uptake include solubilization, endocytosis of drug-CD complexes, API stabilization, or tight junction modulation [51,52,53].

Enhanced oral absorption and oral bioavailability have been reported for PS20 and small molecules [54,55]. PS80 is known for permeability-enhancing properties for small molecules and peptides [37,38,39]. The various derivatives of α-, β-, and γ-CD are widely recognized for enhancing drug absorption [53,56,57], though results vary depending on compound, dose, and model. For example, 10 mg/kg CD improved nasal insulin bioavailability in rabbits from 35% to 69% [35], while 80 mM CD in rats achieved only 4% uptake [58]. A synergistic effect on insulin absorption was also seen when CD was combined with Px188 sublingually [36]. Px188, conversely, has shown reduced uptake of small molecules (e.g., ketoprofen, nadolol) in Caco-2 cells at high concentrations [59].

But can the reported transport results truly be considered reliable when surface-active excipients are present? A key limitation of studies involving excipients is their susceptibility to interfere with the accuracy of many analytical assays, potentially leading to distorted or misleading outcomes. Thus, to verify whether the increased transport in the presence of 10 mg CD is a true result or whether the failed significances of the other excipients are false-negative results, further analyses were performed as summarized in Figure 1: evaluation of excipients’ toxicity on the TMPC barrier, determination of IgG monomer content in the presence of excipients under cell culture conditions, stability of micelle-forming activity of excipients under cell culture conditions and potential of excipients to cross the TMPC barrier and how excipients alter the ELISA readout.

### 3.2. Determination of Excipients’ Toxicity on Cellular Epithelial Barrier Models

We investigated whether excipients in certain concentrations can exhibit toxic effects to epithelial cells and/or loss of barrier integrity in two different cellular airway models (TMPCs and RPMI 2650). Potential cytotoxic effects of all four excipients used here were investigated with Alamar Blue, LDH release, and transepithelial barrier integrity (TEER). TMPCs are primary cells derived from porcine trachea and display many features and functions like in vivo tracheal tissue and form a 3D architecture [1,2]. RPMI 2650 cells are derived from a nasal epithelial cancer and commonly used in pharmacological studies for upper airway delivery [1,7,26,42,60,61]. Toxicity testing is critical for regulatory approval [62]. While Alamar Blue detects mitochondrial activity, LDH measures membrane damage, and TEER evaluates barrier integrity; together, they constitute a comprehensive battery to test potential negative effects on the cells. A 70% viability threshold (ISO 10993-5) for Alamar Blue and LDH release as well as a TEER cutoff of 500 Ω*cm^2^ for TMPCs and of 50 Ω*cm^2^ for RPMI 2450 were used to define cytotoxicity [1,45]. The lower TEER threshold derives from their impaired ability to form adequate barriers [1,2,42].

**Figure 3:**
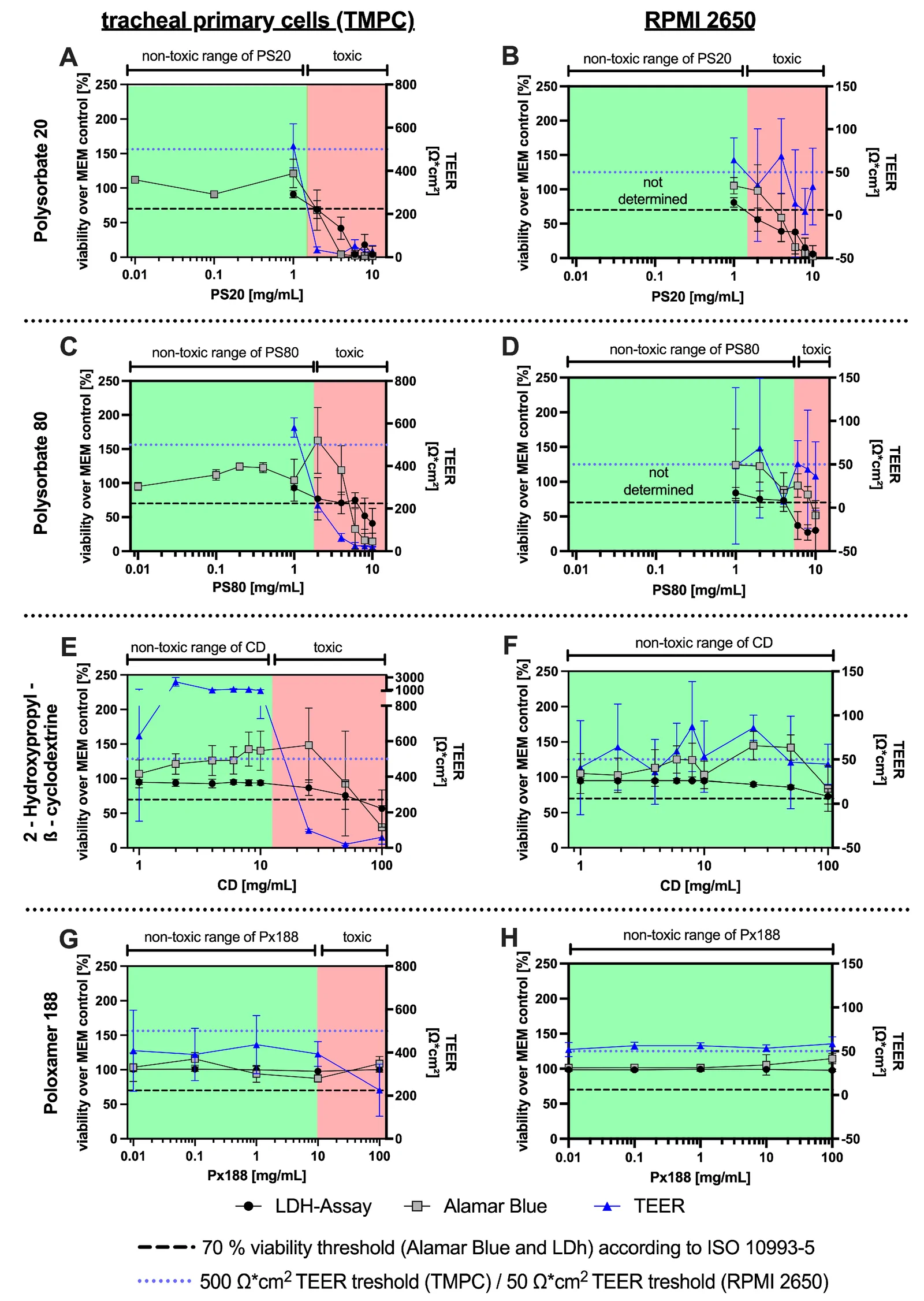
Cytotoxicity of PS20, PS80, CD and Px188 on the two respiratory epithelium in vitro models TMPCs and RPMI 2650. LDH results are displayed in black circles, Alamar Blue results are presented in open grey squares, and TEER results in blue triangles. (**A**,**B**) PS20 demonstrates a clear toxicity beyond 2 mg/mL in both models. (**C**) PS80 toxicity in TMPCs displayed very similar results as PS20 toxicity roughly beyond 2 mg/mL while (**D**) RPMI 2650 needed higher concentrations beyond 5 mg/mL PS80. (**E**) TEER levels dropped significantly at ≥20 mg/mL CD in TMPCs while (**F**) no assay could determine a toxic effect of up to 100 mg/mL CD in RPMI 2650. (**G**) Concentrations beyond 10 mg/mL Px188 had toxic effects in TMPCs, but (**H**) no toxic effects could be observed in RPMI 2650. Non-toxic ranges have a green background; toxic ranges are depicted with a red background. The blue dotted line represents a 500 Ω*cm^2^ threshold. The black dashed line represents the 70% viability threshold according to ISO 10993-5.

The concentrations used to determine the excipients’ toxicity are derived from literature and, hence, lower concentrations were neglected [63,64,65,66]. It should be noted that TEER measurement showed high variability in RPMI 2650, while TEER levels in TMPCs were far more robust and TEER was the most sensitive indicator for all excipients, often detecting barrier disruption before viability loss. High variability and non-predictiveness of TEER measurement of RPMI 2650 are well known in the literature [1,2,61,67,68,69,70]. This immortalized cell line forms a comparably leaky, multi-layered barrier and has demonstrated substantial TEER value dependency on culture conditions, passage number, and experimental protocols [68,70]. Importantly, TEER has been shown to only correlate poorly with permeability in RPMI 2650 cultures. Therefore, TEER should be interpreted cautiously and only in conjunction with functional permeability assays and/or complementary measures of epithelial integrity. Non-toxic concentrations are depicted in Figure 3 with a green background while toxic concentrations show a red background colour.

PS20 demonstrated no negative effects in all three assays when used ≤1 mg/mL (0.298 mg/cm^2^; Figure 3A; ns, *p* ≥ 0.05 in comparison to 70% viability threshold according to ISO 10993-5 in LDH assay; * *p* ≤ 0.05 in comparison to 70% viability threshold in Alamar Blue assay; ns, *p* ≥ 0.05 in comparison to 500 Ω*cm^2^ threshold in TEER assessment) but demonstrated a toxic effect at 2 mg/mL (0.596 mg/cm^2^; **** *p* ≤ 0.0001 for TEER) and beyond in TMPC and RPMI 2650 (Figure 3A,B; AB/LDH: n = 8/10/12, N = 2; TEER: n = 2, N = 1). Thus, in the transport study (Figure 2) a non-toxic concentration of PS20 was used, but 1 mg/mL was at the upper limit.

PS80 revealed a similar cytotoxicity as PS20 in TMPC (Figure 3C; AB/LDH: n = 8, N = 2; TEER: n = 2, N = 1) but was less toxic in RPMI 2650 (Figure 3D; AB/LDH: n = 6/8/10/11, N = 2; TEER: n = 2, N = 1), possibly due to the inconclusive TEER in RPMI 2650. When exposed to TMPC ≥ 2 mg/mL (0.596 mg/cm^2^; **** *p* ≤ 0.0001), the TEER significantly dropped while Alamar Blue viability fell below 70% between 4 and 6 mg/mL (equals 1.19 to 1.79 mg/cm^2^) and LDH viability decreased only at 8 mg/mL (2.38 mg/cm^2^). TEER remained above 500 Ω*cm^2^ only at 1 mg/mL. Thus, the maximum non-toxic PS80 concentration was set to 1 mg/mL, similar to PS20.

CD caused a TEER drop in TMPCs below 500 Ω*cm^2^ at concentrations higher than 10 mg/mL (2.98 mg/cm^2^; *** *p* ≤ 0.001; AB/LDH: n = 8/10/11/12, N = 2; TEER: n = 2, N = 1), although Alamar Blue and LDH assays showed first signs of reduced viability only at 100 mg/mL (29.8 mg/cm^2^; ns, *p* ≥ 0.05; Figure 3E). Interestingly, RPMI 2650 did not display any signs of toxicity over all concentrations investigated (Figure 3F; ns, *p* ≥ 0.05; AB/LDH: n = 6/7/8/9/10, N = 2; TEER: n = 2, N = 1). A total of 10 mg/mL CD used in the transport study with TMPCs was also the maximum non-toxic CD concentration.

Px188 reduced TEER in TMPCs only at 100 mg/mL (29.8 mg/cm^2^; ** *p* ≤ 0.01; AB: n = 2/4, N = 1; LDH/TEER: n = 6/7, N = 2), while Alamar Blue and LDH release did not show any cytotoxicity at any concentrations investigated (Figure 3G; ns, *p* ≥ 0.05; AB/LDH: n = 8, N = 2; TEER: n = 4, N = 2). Likewise, RPMI 2650 demonstrated no signs of cytotoxicity in any of the three tests and their TEER values were stable in the presence of Px188 (Figure 3H). Thus, 10 mg/mL Px188 concentration is the maximum non-toxic concentration.

Determined concentration ranges align with those used in approved products: PS20 (0.1–2 mg/mL), PS80 (0.02–2 mg/mL), and Px188 (0.01–8 mg/mL). For CD, fewer products are on the market (5–330 mg/mL) [12,14,71,72]. Thus, the concentrations used in the IgG transport study can be considered non-toxic for TMPCs, although the higher concentrations are all close to the limit.

The literature reports on excipient cytotoxicity vary depending on assay, cell type, and culture conditions [63,64]. For example, PS20 was more cytotoxic in submerged cultures in comparison to after ALI differentiation, while PS80 toxicity was cell-type dependent [66,73]. Here, the primary TMPC model showed greater sensitivity than RPMI 2650, particularly in Alamar Blue assays.

### 3.3. Excipients’ Potential to Stabilize IgG Under Cell Culture Conditions

Protein aggregation is a complex and variable process influenced by multiple factors, including the specific biopharmaceutical, formulation composition, processing conditions, and the nature and duration of the stress applied [18]. If not optimized, excipients intended to stabilize a formulation may also exhibit insufficient or even pro-aggregating effects [7,15,16,17,18,19]. Further, the presence of aggregates can lead to false-positive ELISA results [74,75]. In our study, this may have resulted in a false-positive effect on IgG transport, although a potential pro-aggregative effect of an excipient was the underlying cause.

**Figure 4:**
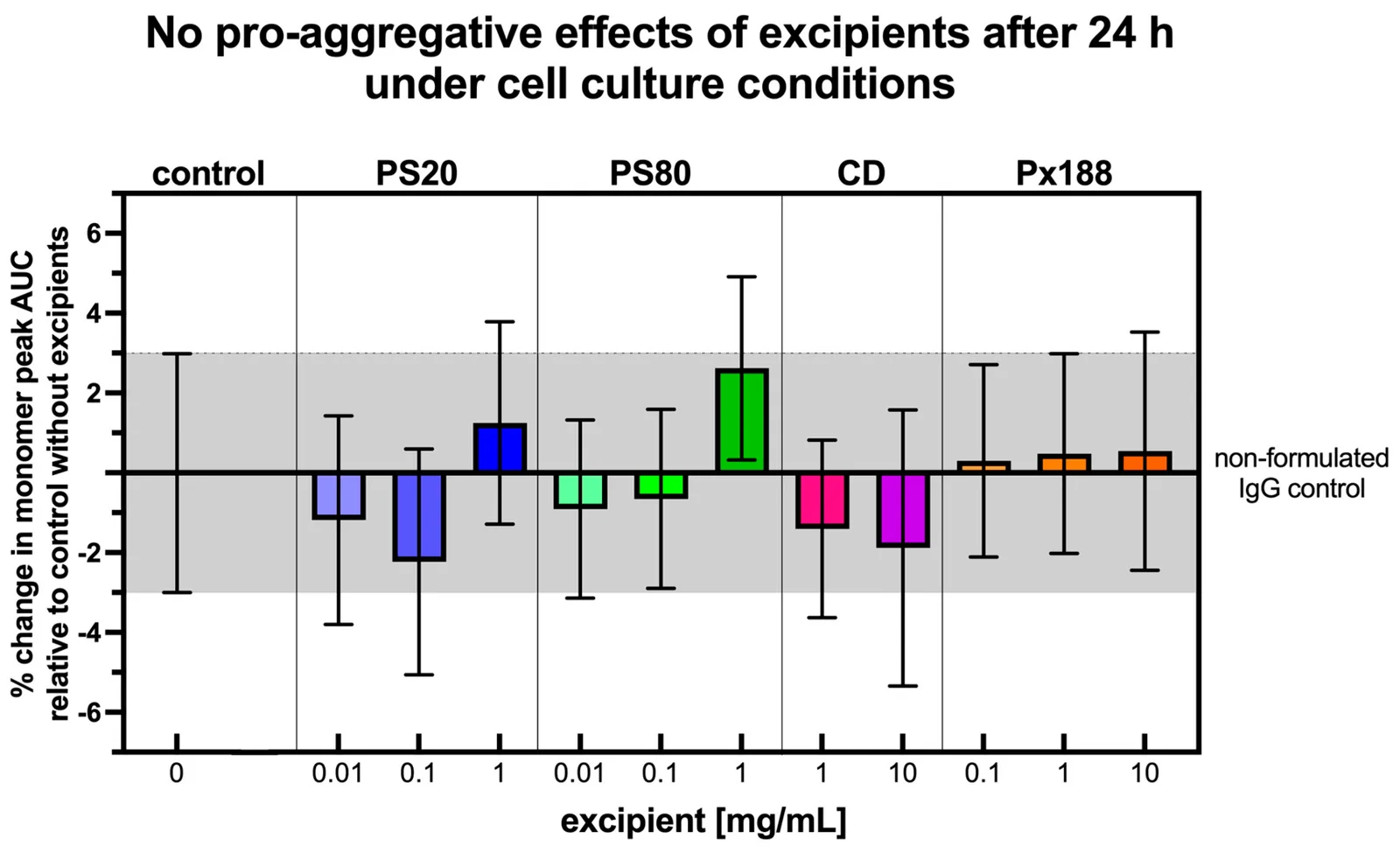
No negative impact on IgG monomer content caused by excipients which could result in biassed outcome in transport studies. IgG monomer peak area changes (in % compared to unformulated controls) in SE-HPLC analysis after 24 h under cell culture conditions (37 °C, 5% CO_2_ and 85% rH; n = 4, N = 2; one-way ANOVA). The mean ± SD of the non-formulated IgG control is depicted as black solid line with a grey background.

Therefore, we quantified here IgG monomers under cell culture conditions, matching the settings and the duration of the transport experiments. Using SE-HPLC, the AUC was analyzed and the monomer peak of an unformulated IgG that was incubated at 37 °C, 85% humidity and 5% CO_2_ in a cell culture incubator for 24 h was used as reference (Figure 4).

The SD of this unformulated sample is depicted as a grey background in Figure 4. Although low concentrations of PS20 (blue bars), PS80 (green bars), and CD (magenta/violet bars) demonstrate a tendency towards a lower monomer content (e.g., negative change in monomer AUC), none of the excipients tested at the different concentrations exhibited a statistically significant change. Since only minor, non-significant changes in monomer content were detected, no significant difference in the number of aggregates should have biassed the transport study. Notably, an increase in monomer AUC—for instance, with 1 mg/mL PS80—may reflect disaggregating effects of certain formulations, a phenomenon previously observed in the literature [76,77]. A representative chromatogram and the monomer AUC values can be found in Supplementary Figure S2 and in Supplementary Table S2.

### 3.4. Excipients’ Micelle-Forming Activity Under Cell Culture Conditions

**Figure 5:**
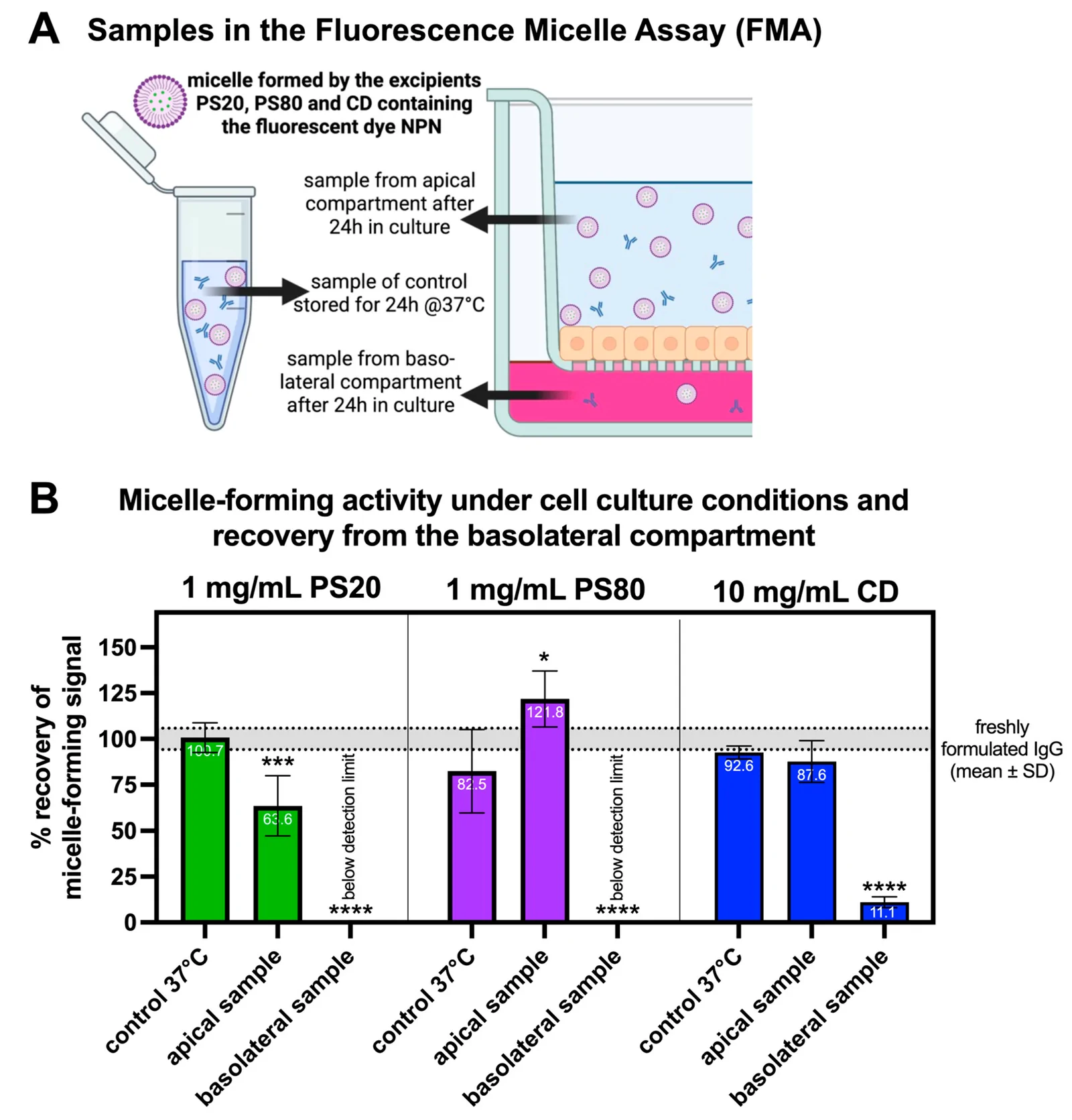
Micelle-forming activity of the excipients PS20, PS80 and CD under cell culture conditions and their ability to cross the epithelial barrier. (**A**) Experimental set-up to determine the recovery of micelle-forming activity in vitro in the presence of primary airway epithelial cells. IgG was formulated with the indicated concentration of excipients and pipetted onto the apical layer of differentiated TMPCs growing on membrane inserts and incubated at 37 °C, 5% CO_2_ and 85% rH for 24 h. A control sample of the formulated IgG was stored at 37 °C for 24 h without exposure to cells. After 24 h, a sample from the apical and a sample from the basolateral compartment were taken and compared to the control sample in a Fluorescence Micelle assay (FMA) using the dye NPN intercalating in micelles formed by excipients. Created in BioRender. Zimmermann, K. (2026) https://BioRender.com/rotigim. (**B**) The data of the FMA are displayed as % recovery of the micelle-forming signal. A freshly formulated IgG control was set to 100% and displayed as grey-shaded area. A non-formulated IgG as a negative control was set to 0%. Both polysorbates PS20 and PS80 remained stable at 37 °C with a higher variability when exposed to the apical surface of the cell barrier, and no micelle-forming activity was detected in the basolateral compartment—suggesting they are either not transported in sufficient levels to be detectable, or their micelles are not stable there. Similarly, CD demonstrated stability of the formed micelles at 37 °C and when exposed to the apical compartment but in contrast to the polysorbates, CD showed a measurable micelle activity (11.1%) in the basolateral compartment. It should be noted that this assay is too insensitive for the lower concentrations of PS20, PS80, and CD and does not work for Px188; n = 2/6, N = 1; values are shown as mean ± SD. Two-way ANOVA with Šidák multiple comparisons; * *p* ≤ 0.05, *** *p* ≤ 0.001, **** *p* ≤ 0.0001.

To determine the micelle-forming activity of the excipients PS20, PS80 and CD under cell culture conditions, an FMA was performed with the samples indicated in Figure 5A. The FMA could not be performed with Px188 as it does not form micelles at 37 °C and, accordingly, no concentration-dependent fluorescence decrease was observed. Previous reports suggest that Px188 undergoes self-assembly in a manner that interferes with dye incorporation required for the FMA [12,78,79,80,81].

The micelle-forming activity is displayed as % recovery from a freshly formulated IgG control (set to 100%) and a non-formulated IgG as a negative control (set to 0%). Cell culture conditions contain exposure for 24 h to 37 °C, 5% CO_2_, and 85% rH (control 37 °C), the above-mentioned conditions including exposure to the apical TMPC surface (apical sample) or even transepithelial transport to the basolateral side (basolateral sample). Cell culture medium in the basolateral side did not influence the FMA. However, the FMA turned out to be rather insensitive in this study with a sensitivity ≥ 0.3 mg/mL micelle-forming agents. Therefore, the excipients at concentrations of 0.01 mg/mL PS20, 0.1 mg/mL PS20, 0.01 mg/mL PS80, 0.1 mg/mL PS80, but also 1 mg/mL CD (due to too-high deviations) could not be meaningfully quantified. Likewise, samples containing 0.01 mg/mL PS20 or PS80 could not be resolved by FMA as previously described [82,83,84].

While the polysorbate control samples stored at 37 °C in the incubator were not significantly altered from their positive control, the CD control at 37 °C was a bit lower indicating a small loss of micelles under these conditions. Exposure to cells in the apical compartment significantly altered the recovery of micelle-forming activities for both polysorbates compared to their individual control at 37 °C (*** *p* = 0.001 for PS20 and **** *p* ≤ 0.0001 for PS80, but not for CD (all N = 6, n = 2; two-way ANOVA with Dunnett’s multiple comparison). Very interestingly, no micelle-forming activity was recovered from the basolateral compartments when 1 mg/mL PS20 or 1 mg/mL PS80 was present in the apical compartment, indicating either that no micelles are transported to the basolateral compartment or that micelles, even if transported here, are not stable under these conditions over 24 h. Only in cells treated with an IgG formulated in 10 mg/mL CD, 11.1% of the micelle-forming activity was recovered from the basolateral compartment, indicating that up to 1.11 mg/mL micelle-forming agents are present in this compartment. As stated above, this CD concentration is at the borderline to the sensitivity limit of the FMA. Therefore, this observation was approximated to 1 mg/mL CD in further analyses as the exact amount of CD recovered from the basolateral compartment was expected to be in this order of magnitude.

Conclusively, this indicates that the presence of PS20 and PS80 in basolateral samples is not relevant and, thus, is not likely to impact quantification with ELISA. However, CD has the potential to form micelles also in the basolateral compartment and therefore an interference with ELISA quantification cannot be excluded.

### 3.5. Matrix Effects of Excipients on ELISA and Its Relevance for the Readout of Transport Studies

**Figure 6:**
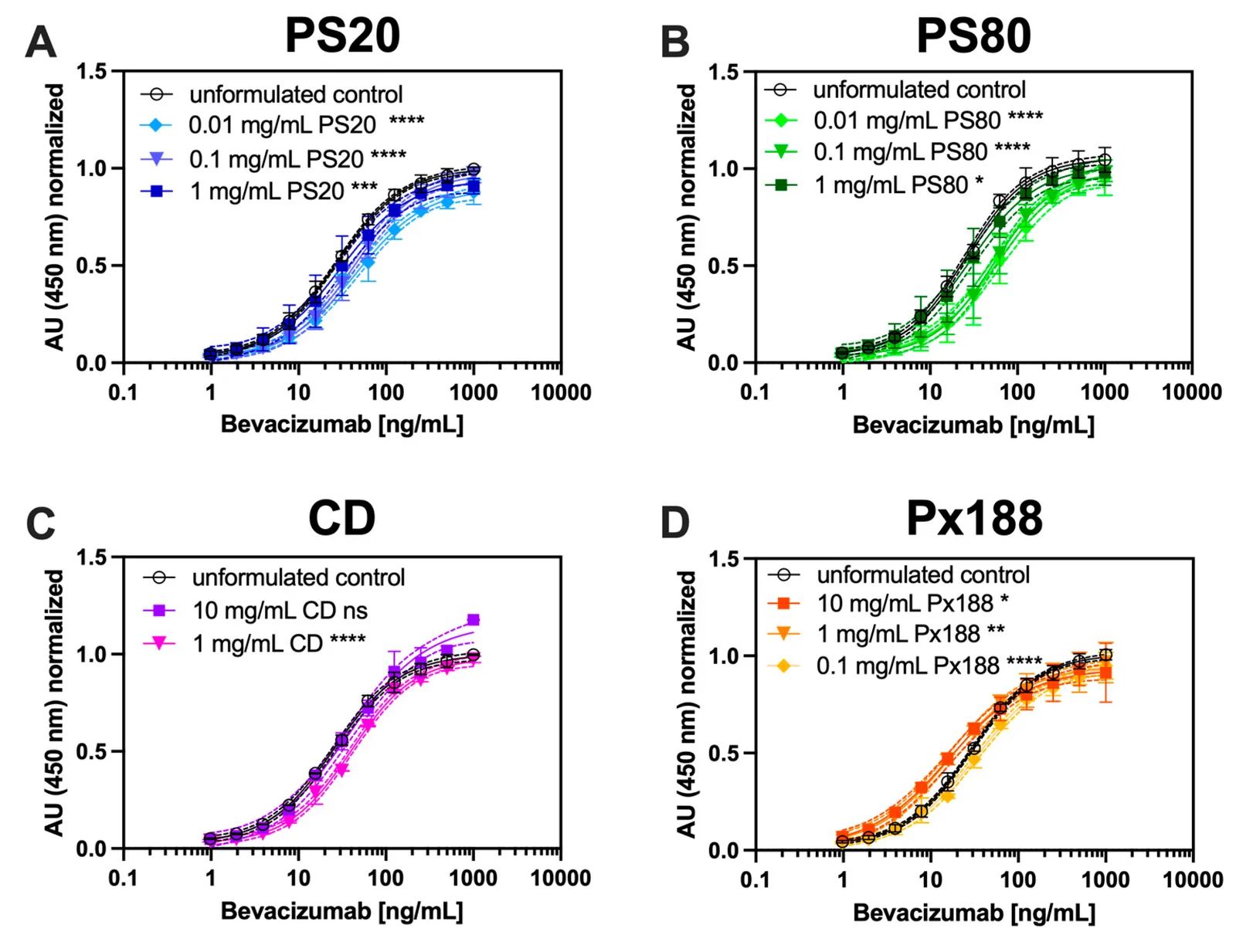
Matrix effects of excipients on ELISA calibration curves and thereby on IgG quantification. (**A**) Calibration of 1, 0.1 and 0.01 mg/mL PS20 (coloured in blue) compared to IgG calibrator titrated in MEM without any excipient (black open circles), n = 2, N = 1. (**B**) Similar results are obtained from the calibration curves of 1, 0.1 and 0.01 mg/mL PS80 (coloured in green) compared to IgG calibrator without any excipient (black open circles), n = 2, N = 1. (**C**) While the calibration with 1 mg/ml CD (coloured magenta) is significantly right-shifted compared to the calibration without any excipients (black), there is no significant (ns) difference between 10 mg/mL CD (violet) and the unformulated control, n = 2, N = 1. (**D**) Standard calibration curve of 10, 1, and 0.1 mg/mL Px188 (yellow—orange—red) compared to the unformulated control (black). Likewise, the effect is more pronounced for the lower concentrations. But interestingly, lower concentrations of Px188 result in a right-shift in the calibration curves, n = 2, N = 1. All data are shown as mean ± SD. Ordinary two-way ANOVA was performed for comparing the calibration curves to the unformulated standard without excipients. *p* > 0.05, not significant (ns); * *p* ≤ 0.05; ** *p* ≤ 0.01; *** *p* ≤ 0.001; **** *p* ≤ 0.0001.

It is well accepted that formulations significantly influence protein folding and thereby functionality, particularly antigen binding of antibodies [85]. In addition, matrix effects of excipients are well known and are challenging for the analytics of biopharmaceuticals [48,86,87]. To assess the potential effects of excipients present in the basolateral compartment, ELISA standard curves were compared in the presence and absence of excipients. Thus, in contrast to the analysis shown in Figure 2, the calibrator IgG was diluted in MEM medium with and without excipients. All other parameters were followed to the standard protocol of the sandwich ELISA used to analyze the data in Figure 2. The results are summarized in Figure 6.

Figure 6A,B demonstrates that polysorbate-containing samples, those with 1 mg/mL CD (Figure 6C) and those formulated with 0.1 mg/mL Px188 (Figure 6D) produced a rightward shift in the calibration curves and—except for 1 mg/mL CD—also lower signal, corresponding to a reduced assay sensitivity when compared to the non-formulated IgG calibrator. In contrast, Px188 at 1 and 10 mg/mL caused a leftward shift, suggesting higher IgG levels. Interestingly, the effect on the ELISA is more pronounced for the lower concentrations of all tested excipients. Accordingly, no significant effect compared to the calibrator in the absence of excipients was observed for CD at 10 mg/mL (*p* ≥ 0.05). These results confirm that excipients can affect analytical accuracy and consequently the quantification of analyzed samples, making their interpretation challenging. Rightward shifts suggest underestimation of IgG concentrations, while leftward shifts may lead to overestimation if calibration curves without excipients are used. Intra-assay variability was lowest for CD and Px188, while PS20 and PS80 showed greater day-to-day variation.

As demonstrated in Figure 5B, only the highest concentration of CD (10 mg/mL) resulted in detectable levels of micelles in the basolateral compartment quantified by FMA. Since the detected basolateral concentration of micelle-forming CD was approximately 1 mg/mL, the effects on IgG quantification were evaluated in the absence and in the presence of 1 mg/mL CD in the basolateral MEM medium (Supplementary Figure S3).

Figure S3A shows the effect of quantifying the basolateral samples with a matrix-matched calibration curve containing 1 mg/ml CD (coloured magenta; interpolated concentrations are indicated in green squares) or with an unformulated calibration curve (coloured in black; interpolated concentrations are indicated in yellow circles). As the CD-containing (magenta) calibration is right-shifted, the interpolated concentrations are higher than for the unformulated calibration (black). After correction for dilution and basolateral volume, this results in a 1.2-fold higher total mass of 4135.5 ± 1967.25 ng IgG recovered from the basolateral compartment and quantified with 1 mg/mL CD-containing calibrator while only 3422.1 ± 1482.39 ng are quantified with the unformulated non-matrix-matched calibrator. Normalized to the permeability of an unformulated IgG in the absence of any excipients (100.0 ± 49.60%) this corresponds to 349.8 ± 166.39% quantified with the 1 mg/mL CD-containing calibrator vs. 289.4 ± 125.38% quantified with the unformulated control (n = 6, N = 3; Figure S3B). Due to the high standard deviations, this difference is not statistically significant; thus, the observed matrix effects do not impact the outcome of the IgG transport across the mucosal airways investigated here.

## 4. Discussion

Pharmaceutical excipients play a crucial role in the formulation of drug candidates, aiming to improve ease of administration, enable targeted delivery such as inhalation, and ensure optimal bioavailability [48]. In particular, pharmaceutical excipients are essential for the successful formulation of protein aerosols, where they stabilize the biopharmaceutical protein and ensure efficient pulmonary delivery. Surfactants such as polysorbates and poloxamers, but also cyclodextrins, protect proteins from interfacial stress and aggregation during nebulization, mitigating physical and chemical stresses while enabling appropriate aerodynamic properties for inhalation [88]. A major driver of aggregation during aerosolization is exposure to the air–liquid interface, which is considered one of the most critical contributors to aggregation in sprays and nebulizers [7], although shear and extensional stresses also play a role. The resulting exposure of hydrophobic regions and aggregation-prone sequences facilitates protein–protein association, especially under conditions of high local concentration [89]. This underscores the need for inhalation-specific formulations with suitable excipients and for careful selection and characterization of nebulization devices [7,18,49].

Despite the doubtless protective role of excipients in protein aerosols, excipients may also influence biological interactions and analytical readouts, highlighting the need for studies like the one presented here.

The effects of cyclodextrins on uptake and adsorption are well described for small molecule drugs, peptides and proteins, potentially mediated by endocytosis of complexes, and tight junction modulation [7,35,36,50,51,52,53,90]. Likewise, polysorbates and poloxamers have been linked to enhanced absorption and bioavailability of small molecules and peptides by improving transport capacity [37,38,39,59,91]. Although more focus is placed on the underlying mechanisms [92], the fact that excipients also provide several other functions—many of them interfering with bioanalytical assays—is still insufficiently developed [48].

A potential cytotoxicity of excipients would distort uptake studies at epithelial barriers by loss of barrier integrity with detached cells and disrupted or non-functional tight junctions, dysregulation of transporters and metabolism resulting in loss of active transport [93]. In this context, the neonatal Fc receptor (FcRn) plays a pivotal role in regulating IgG transport across epithelial barriers [2,25,26,49,94]. However, excipient interactions with the protein’s Fc or Fab region may also alter FcRn binding, potentially impacting transcytosis efficiency. Finally, damaged membranes may expose hydrophobic surfaces that can increase unspecific binding and absorption of the delivered proteins [95,96]. Therefore, the underlying mechanism by which excipients, specifically CD, affect IgG transcytosis requires further studies. For example, even though we could demonstrate barrier integrity after 24 h for the selected excipient concentrations, it may be of great interest whether the observed effects are attributable to a reversible modulation of the epithelial barrier, for example, through changes in tight junction organization, or to a direct effect on IgG–FcRn interactions and consequently FcRn-mediated transcytosis. Such experiments would help to distinguish between a general increase in paracellular permeability and a specific modulation of receptor-mediated IgG transport and thereby provide a mechanistic basis for the excipient-dependent effects observed in this study.

Although excipients should prevent aggregation and stabilize the protein, if not optimized, excipients may also exhibit insufficient or even pro-aggregating effects [7,15,16,17,18,19]. In such aggregates, epitopes can become buried or conformationally altered, so that quantification by ELISA is impaired because of less efficient binding of capture or detection antibodies; thus, an apparent underestimation of the analyzed protein may occur, but also an overestimation if disruption of aggregates is triggered by excipients [97]. Therefore, an orthogonal SEC assay to determine the monomer content was used here to uncover any potential anti- or pro-aggregative effects under cell culture conditions. Notably, an increase in monomer AUC—for instance, with 1 mg/mL PS80—may reflect disaggregating effects of certain formulations, a phenomenon previously observed in the literature [76,77]. Polysorbate stability and degradation are major concerns in pharmaceutical research and their stability under conditions suitable for culturing epithelial cells with continuous exposure to oxygen at 37 °C is not sufficiently explored [98]. It is well known that excipients such as polysorbates can undergo hydrolysis by cellular enzymes leading to their degradation and that can affect protein stability [99]. Further, oxidation of polysorbates may occur during cell culture at elevated temperature and by exposure to oxygen and light [100,101]. Thus, after excluding potential effects of excipients on the IgG quality under cell culture conditions, it shall be verified that the activity of excipients to form micelles is retained under cell culture conditions. Although polysorbates are common in formulations, CD is emerging as an alternative with fewer stability concerns [102]. Originally used to enhance solubility and stability of small molecules, CD also reduces protein aggregation [103,104]. This effect arises from interactions with aromatic residues Phenylalanin, Tryptophan and Tyrosin or from displacing proteins from interfaces [82,105]. Similar to polysorbates, multiple detection and quantification techniques have been developed for CD [98,106].

Excipients in IgG formulations interact with the protein at its surface, at interfaces, in the bulk solution, but also within micelles. Nonionic surfactants form micelles above the critical micelle concentration (CMC), which fundamentally changes how the surfactant is distributed between interfaces, bulk monomer, and micellar aggregates [107]. Therefore, the micelle formation enabled by polysorbates and CD was investigated under cell culture conditions. Methods like FMA are simple, low-cost, and robust, but quantify surfactants only above their CMC, reported as 0.06 mg/mL for PS20, ~0.015 mg/mL for PS80, and 5 mg/mL for CD [82,83,84]. Our data for polysorbates at 0.01 mg/mL were comparable to the above-mentioned reports and below the detection limit. Furthermore, 1 mg/mL CD was borderline to detect with too high variation for a meaningful analysis; thus, the recovered basolateral concentration of 1.11 mg/ml was approximated to 1 mg/mL in further analyses. Increases in fluorescence signal of small molecules after the complexation with CD have been described [108,109]. Increased fluorescence due to NPN dye complexation with CD’s hydrophobic core may explain why FMA was applicable even below CD’s CMC, though with high variability [110,111]. To our knowledge, this approach has not yet been used for CD quantification in pharmaceutical formulations.

FMA does not distinguish between intact polysorbates and degradation products and has varying sensitivity depending on hydrophobicity [112]. It remains robust in protein-rich environments (up to 75 mg/mL protein; [113,114]). However, future work incorporating chromatographic separation could clarify whether stable fluorescence signals reflect true lack of excipient uptake or assay limitations [115,116].

In the case of Px188, self-assembly of the excipient has been repeatedly reported [78,79,80]. This observation makes methods that are based on the principle of dye incorporation difficult to implement, as the interaction of the substances may vary [12]. Additionally, various utilized analytical methods, possible surface tension-lowering impurities, a high(er) molecular weight (in comparison to, e.g., polysorbates), and polymeric characteristics result in a massive discrepancy in reported CMC values of Px188, ranging from 0.0055% (*w*/*v*) up to over 10% (*w*/*v*) [12]. Degradation of Px188 via oxidation has been described [117]. Therefore, new methods are under development to evaluate its suitability as a PS alternative [89,118,119]. However, data on Px188 and its degradation products’ effects on protein stability remain limited [12].

The FMA is a rapid and easy format but with the above-detailed limitation in sensitivity. Therefore, future studies should use alternative analytical strategies such as liquid chromatography with charged aerosol detection or evaporative light-scattering detection quantifying the surfactants directly and that are largely independent of their micellar microenvironment [120]. In addition, simultaneous quantification of PS20 and Px188 is feasible [121]. Moreover, reversed-phase liquid chromatography coupled with mass spectrometry will allow highly sensitive, selective quantification of polysorbate constituents at sub-ppm levels in complex protein matrices [122]. Such methodology will support studies to understand whether CD mediates the drug uptake or whether CD is also adsorbed during drug uptake.

Generally, micelle formation is important for biopharmaceuticals primarily because it sets the free surfactant monomer activity that controls interfacial coverage, and can solubilize co-formulated small hydrophobic molecules; but micelles are not generally thought to form strong, stoichiometric complexes with IgG in standard formulations [107].

It should be noted that newer studies have determined that micelles of IgG formulated with polysorbates are relatively small in the low nm-range and that the stabilizing effect in typical monoclonal antibody formulations might be unlikely to be dominated by stable protein–micelle complexes [13]. Instead, stabilization might be due to surfactant adsorption to interfaces and weak, nonspecific binding to hydrophobic protein patches, with micelles mainly acting as a reservoir that buffers the free monomer concentration [123].

Finally, excipients can influence study outcomes through bioanalytical matrix effects because excipients are often present in study samples but absent from calibration standards; thus, they may lead to over- or underestimation of drugs during quantification [48]. This highlights the need to better understand the mechanisms underlying excipient-driven matrix effects and to develop strategies to mitigate them.

The observed impact of excipients on ELISA readout highlights the underappreciated influence of formulation, particularly surfactants, on biopharmaceutical performance from production to analytical evaluation. While many studies have focused on the excipient-dependent stabilization of biopharmaceuticals [7,76], few have addressed the need to adapt analytical methods to such formulation effects [40,41]. In the sandwich ELISA used here, both the capture and detection antibodies are directed against the Fc domain of human IgG; therefore, the assay signal depends on the Fc domain being properly folded and accessible. Previous findings provide support for the effects observed in our study. PS20, for example, enhanced antibody binding in immunoblotting, potentially through protein refolding [33], but has also been reported to elute adsorbed antigens or interfere with ELISA analyses [33,34]. Similarly, PS80 altered the SE-HPLC elution profiles of different erythropoietin forms, although its impact on ELISA performance was not fully investigated [41]. Singh et al. reported that polysorbates preferentially interact with the Fab region, presumably due to exposed hydrophobic patches [85]. Consistent with a potential effect of polysorbates on antibody–antigen interactions, in our recent study, PS80 increased IgG binding to S. pneumoniae antigens in a multiplex bead assay, although this effect was reduced following aerosolization [49]. In contrast, other studies have reported minimal interactions between polysorbates and immunoglobulins, while observing moderate binding to albumin [124]. Given that polysorbates exhibited the highest ELISA variability in the present study, future investigations should determine whether changes in polysorbate stability over time contribute to the observed analytical inconsistencies.

Data on how CD interferes with ELISA are limited. It has known stabilizing effects and may displace proteins from interfaces or be used as an eluent in hydrophobic charge-induction chromatography [125,126,127].

Px188 may alter analyte conformation (e.g., cardiac troponin) but not antibody binding [128]. It can also displace adsorbed antibodies and affect IgG conformation or orientation [129,130]. Interestingly, Px188 combined with poloxamer 407 enhanced IgG avidity in an HIV vaccine model [40].

Beyond their stabilizing functions, we demonstrated in this study that commonly used excipients in protein formulations for respiratory drug delivery can influence ELISA accuracy. Our results and findings previously published from other research groups underscore the importance of validating each analytical method with respect to potential formulation-related effects. That is relevant if excipients cross the epithelial barrier and are present in the samples to be quantified by ELISA. Using a non-matrix-matched calibration, IgG transcytosis was increased by 2.9-fold in the presence of 10 mg/mL CD compared to an unformulated control while 3.5-fold was determined with a matrix-matched calibration. The difference between the two results lacks statistical significance also due to the rather high standard deviations of transcytosis experiments (unpaired Welch’s *t*-test; 289.4 ± 125.4% vs. 349.8 ± 166.4%; mean difference = 60.34 percentage points, 95% CI −131.1 to 251.8; Welch’s *t*-test, *p* = 0.4955). Taken together, these findings support a CD-associated increase in transport. However, the matrix-matched experiments alone cannot definitively exclude a contribution from matrix effects because of the limited sample size and variability. Collectively, these findings emphasize that excipient effects on analytics and biopharmaceutical activity vary depending on methodology, choice of biopharmaceutics, concentration and quality of API and excipients. Thus, their influence should be evaluated case by case.

## 5. Conclusions

Surface-active excipients can significantly influence the interpretation of transepithelial IgG transport by affecting cell viability, barrier integrity, micelle formation, and ELISA-based quantification, creating potential for both false-positive and false-negative results. Under the conditions tested, the increased IgG transport observed with 10 mg/mL CD in a primary model from respiratory mucosa (TMPC) cannot be explained by excipient toxicity, IgG aggregation, or analytically relevant matrix effects alone and thus likely reflects a true CD-mediated enhancement of IgG transport. Nonetheless, rigorous methodological controls for excipient-related artefacts are essential when assessing mucosal antibody delivery in vitro.

### Limitations

As discussed above, the study has its limitations in the FMA assay’s sensitivity for the quantification of excipients.

## Data Availability

Data are contained within the article and Supplementary Material.

## Supplementary Materials

The following supporting information can be downloaded at: https://www.mdpi.com/article/10.3390/pharmaceutics18101262/s1, Table S1: Osmolality of the excipients’ solution; Figure S1: Absolute effects of the excipients PS20, PS80, CD and Px188 on transepithelial transport of IgG; Figure S2: Representative chromatogram of SE-HPLC analysis of PS80 samples; Table S2: SE-HPLC data of IgG monomer integration of the excipients’ samples; Figure S3: Influence of matrix-matched calibration on the quantification of basolateral IgG samples.

## Abbreviations

The following abbreviations are used in this manuscript:

ALI	Air–Liquid Interface
API	Active Pharmaceutical Ingredient
AUC	Area under the curve
CD	2-Hydroxypropyl-β-cyclodextrin
CMC	Critical micelle concentration
DMEM	Dulbecco’s Modified Eagle Medium
DMSO	Dimethyl Sulfoxide
EBSS	Earl’s Balanced Salt Solution
EDTA	Ethylenediaminetetraacetic acid
ELISA	Enzyme-linked immunosorbent assay
Fab	Antigen-binding Fragment
FBS	Fetal Bovine Serum
FMA	Fluorescence Micelle Assay
HPLC	High-Performance Liquid Chromatography
IgG	Immunoglobulin G
INT	Iodonitrotetrazolium
ISO	International Organization for Standardization
LDH	Lactate Dehydrogenase
MEM	Eagle’s Minimum Essential Medium
NEAA	Non-essential amino acids
NPN	N-phenyl-1-naphtylamine
PBS	Phosphate-buffered Saline
PS20	Polysorbate 20
PS80	Polysorbate 80
rH	Relative humidity
SEC	Size-Exclusion Chromatography
SE-HPLC	Size-exclusion–high-performance liquid chromatography
TEER	Transepithelial electrical resistance
TMPCs	Tracheal Mucosa Primary Cells
w/o	Without
