# Supplementary material for: Airway Transepithelial Transport of IgG Formulated with Excipients: A Case Study on What to Consider Regarding Impact on Epithelial Barrier Integrity, IgG Quality and Matrix Effects During the Analytics
Source: Pharmaceutics. 2026 Oct 6;18(10):1262. doi: 10.3390/pharmaceutics18101262 (PMC13708301; doi:10.3390/pharmaceutics18101262)
Supplement: Supplementary file 1 [file pharmaceutics-18-01262-s001.zip › pharmaceutics-4567129-supplementary.pdf]

Supplementary Material

Table S1: Osmolality of the excipients' solution

Concentration [mg/mL]	Osmolality [mOsm/kg] mean	Osmolality [mOsm/kg] ±SD
	**Polysorbate 20 solutions in PBS pH 7.4. N = 2**	
10	0.285	0.003
8	0.278	0.004
6	0.277	0.002
4	0.276	0.004
2	0.276	0.002
1	0.272	0.002
	**Polysorbate 80 solutions in PBS pH 7.4. N = 2**	
10	0.281	0.000
8	0.276	0.003
6	0.277	0.002
4	0.278	0.002
2	0.278	0.003
1	0.279	0.004
	**2-Hydroxypropyl-β-cyclodextrine solutions in PBS pH 7.4. N = 2**	
10	0.281	0.000
8	0.276	0.003
6	0.277	0.002
4	0.278	0.002
2	0.278	0.003
1	0.279	0.004
	**Poloxamer 188 solutions in PBS pH 7.4. N = 2**	
10	0.251	0.000
1	0.277	0.001
0.1	0.278	0.002
0.01	0.277	0.001

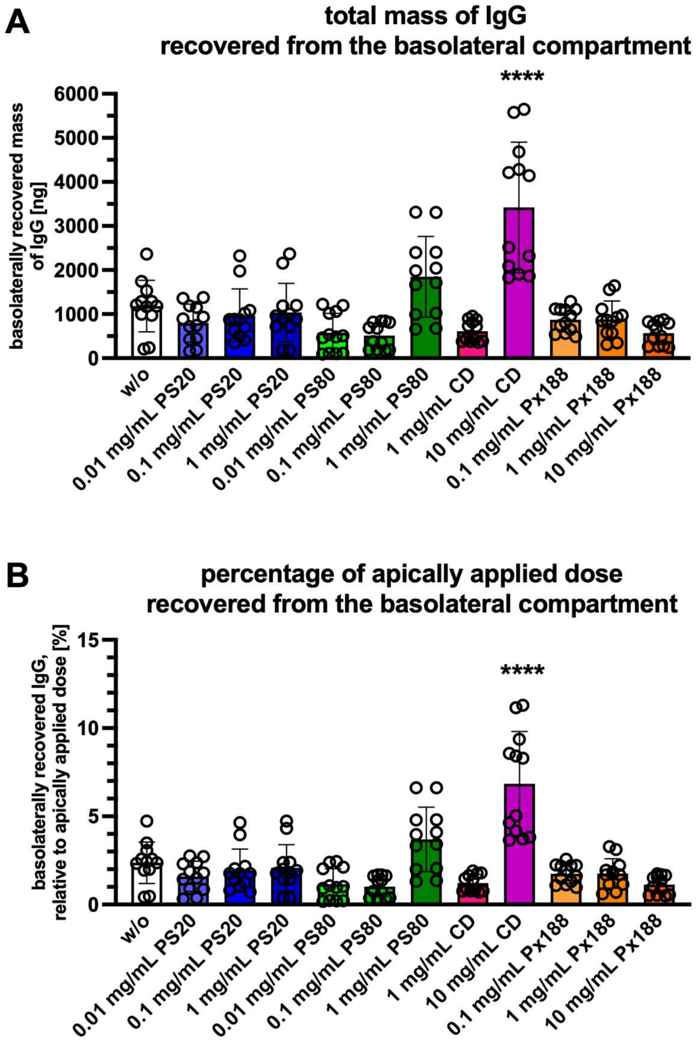

Figure S1: **Absolute effects of the excipients PS20, PS80, CD and Px188 on transepithelial transport of IgG.** Excipients were used in the indicated concentrations [mg/mL]. Only 10 mg/mL CD increased the IgG transport significantly (****$p \leq 0.0001$; one-way ANOVA with Dunett's multiple comparison), all other different concentrations of the excipients failed clearly statistical significance. All data are displayed as mean ± SD. **A)** Total amount of IgG that was recovered from the basolateral compartment. **B)** Basolaterally recovered amount of IgG expressed as percentage of apically applied dose.

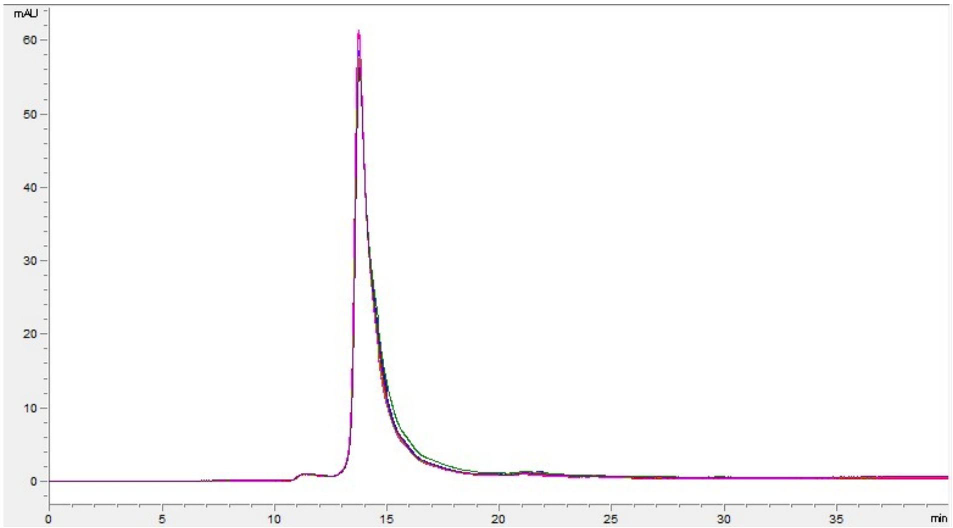

Figure S2: Representative chromatogram of SE-HPLC analysis of PS80 samples

Table S2: SE-HPLC data of IgG monomer integration of the excipients' samples. 0h indicates freshly diluted samples.

**PS20 samples**; n = 4, N = 2						
excipient's concentration	1 mg/mL		0.1 mg/mL		0.01 mg/mL	
time incubated	0h	24h	0h	24h	0h	24h
AUC mean [mAU*s]	3581.83	3604.20	3557.23	3455.55	3419.65	3357.70
AUC SD [mAU*s]	81.08	70.69	60.30	94.20	40.05	74.35
% (mean)		0.62		-2.86		-1.81
%(SD)		1.97		2.65		2.17
**PS80 samples.** n = 4, N = 2						
excipient's concentration	1 mg/mL		0.1 mg/mL		0.01 mg/mL	
time incubated	0h	24h	0h	24h	0h	24h
AUC mean [mAU*s]	3575.50	3646.78	3489.53	3444.88	3338.15	3286.80
AUC SD [mAU*s]	88.20	45.00	63.07	37.05	7.12	33.50
% (mean)		1.99		-1.28		-1.54
%(SD)		1.26		1.06		1.00
**CD samples.** n = 4, N = 2						
excipient's concentration	10 mg/mL		1 mg/mL		without excipients	
time incubated	0h	24h	0h	24h	0h	24h
AUC mean [mAU*s]	3296.20	3213.50	3386.05	3317.18	3255.85	3235.45
AUC SD [mAU*s]	175.89	127.61	29.95	32.72	99.78	97.45
% (mean)		-2.51		-2.03		-0.63
%(SD)		3.87		0.97		2.99
**Px188 samples.** n = 4, N = 2						

excipient's concentration	10 mg/mL		1 mg/mL		0.1 mg/mL	
time incubated	0h	24h	0h	24h	0h	24h
AUC mean [mAU*s]	3448.85	3445.93	3555.90	3550.65	3541.43	3529.80
AUC SD [mAU*s]	106.60	102.73	102.60	67.20	63.14	57.57
% (mean)		-0.08		-0.15		-0.33
%(SD)		2.98		1.89		1.63

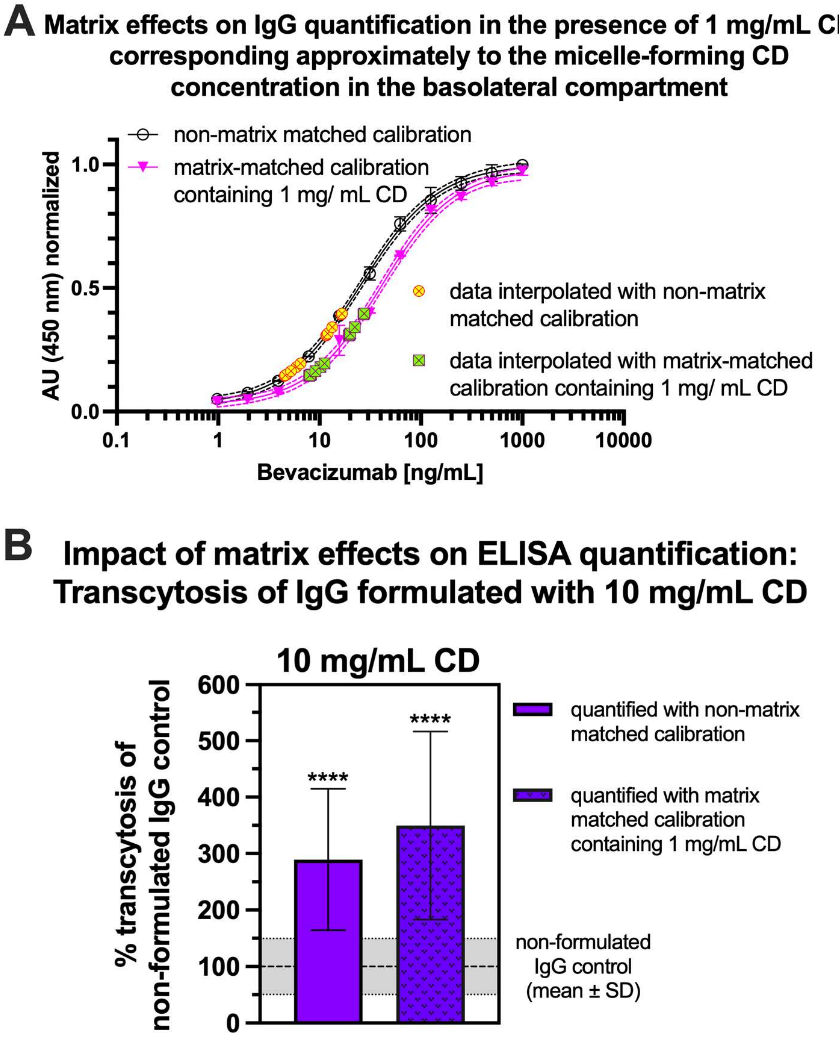

Figure S3: Influence of matrix-matched calibration on the quantification of basolateral IgG samples. A) Basolateral concentrations were determined using either a matrix-matched calibration curve containing 1 mg/mL CD (magenta; interpolated concentrations shown as green squares) or an unformulated non-matrix matched calibration curve (black; interpolated concentrations shown as yellow circles). The CD-containing calibration curve is right-shifted, resulting in higher interpolated concentrations compared to the unformulated calibration. **B)** When normalized to the permeability of unformulated IgG in the absence of excipients (set 100.0% ± SD depicted as grey background), this equals an apparent 1.2-fold higher recovery for the matrix-matched CD-containing calibration versus

the non-matrix matched control ($n = 6$, $N = 3$). Despite this trend, the difference is not statistically significant due to high variability (unpaired Welch's t test: ns, $p > 0.05$, 289.4 ± 125.4% vs. 349.8 ± 166.4%; mean difference = 60.34 percentage points, 95% CI −131.1 to 251.8; Welch's t-test, $p = 0.4955$), indicating that the observed matrix effects do not meaningfully impact the overall interpretation of IgG transport across the mucosal airway model. ****$p \leq 0.0001$ compared to non-formulated control; one-way ANOVA with Dunett's multiple comparison). All data are displayed as mean ± SD.
